# Assessing Regional Ecosystem Conditions Using Geospatial Techniques—A Review

**DOI:** 10.3390/s23084101

**Published:** 2023-04-19

**Authors:** Chunhua Zhang, Kelin Wang, Yuemin Yue, Xiangkun Qi, Mingyang Zhang

**Affiliations:** 1 Department of Biology, Algoma University, Sault Ste. Marie, ON P6A2G4, Canada; chunhua.zhang@algomau.ca; 2 Key Laboratory for Agro-Ecological Processes in Subtropical Region, Institute of Subtropical Agriculture, Chinese Academy of Sciences, Changsha 410125, China; 3 Huanjiang Observation and Research Station for Karst Ecosystem, Chinese Academy of Sciences, Huanjiang 547100, China

**Keywords:** ecosystem conditions, regional assessment, landscape pattern, remote sensing, spatial big data

## Abstract

Ecosystem conditions at the regional level are critical factors for environmental management, public awareness, and land use decision making. Regional ecosystem conditions may be examined from the perspectives of ecosystem health, vulnerability, and security, as well as other conceptual frameworks. Vigor, organization, and resilience (VOR) and pressure–stress–response (PSR) are two commonly adopted conceptual models for indicator selection and organization. The analytical hierarchy process (AHP) is primarily used to determine model weights and indicator combinations. Although there have been many successful efforts in assessing regional ecosystems, they remain affected by a lack of spatially explicit data, weak integration of natural and human dimensions, and uncertain data quality and analyses. In the future, regional ecosystem condition assessments may be advanced by incorporating recent improvements in spatial big data and machine learning to create more operative indicators based on Earth observations and social metrics. The collaboration between ecologists, remote sensing scientists, data analysts, and scientists in other relevant disciplines is critical for the success of future assessments.

## 1. Introduction

Regional spatial and temporal variation necessitate the continuous monitoring and assessment of ecosystem conditions. However, the spatial complexity and dynamic properties of ecosystems make it challenging to monitor ecosystem conditions accurately. A region, in many cases, is a mosaic with a variety of ecosystems (e.g., forest, wetlands, lakes, and rivers), each comprising of numerous components (e.g., trees, grasses, and animals). Ecosystem conditions change constantly due to natural dynamics and the impacts from climatic change, invasive species, pollution, habitat alteration, and many other anthropogenic disturbances. This adds to the challenges of comprehensively and continuously evaluating ecosystem conditions.

Ecosystem conditions may be monitored and assessed from one or more perspectives: structure (e.g., species composition), function (goods and services), and/or processes (e.g., photosynthesis, evapotranspiration, primary productivity, and nutrient cycling) [1,2]. While assessment results from monitoring and assessing of one or several variables relevant to the regional ecosystem can indicate past ecosystem conditions, it is likely that such assessments may not provide a full picture. As a result, there have been efforts to assess ecosystems from numerous, selected perspectives to achieve a full picture of the status and degree of degradation at regional scales.

The complex nature and large spatial variation of regional ecosystems frequently dictate that multiple variables with a spatial dimension need to be integrated for an effective assessment. Geospatial techniques, mainly Geographic Information Systems (GIS), have contributed significantly to the collection and analysis of spatial information for regional environments [3,4]. Map layers of different features of the regional ecosystem (or variables) can be collected, stored, integrated, and assessed using GIS.

The most common means of comprehensively assessing ecosystem conditions using geospatial techniques in the last 20 years are ecosystem service assessments. These assessments followed the Millennium Ecosystem Assessment, started in 2001, the Aichi Biodiversity Targets in 2010, the establishment of the Intergovernmental Platform on Biodiversity and Ecosystem Services in 2012, and the Post-2020 Global Biodiversity Framework [5,6]. The principal approaches for regional ecosystem service assessments have been benefit transfer, ecological production function modeling, extrapolation, and data integration [7]. Note that the original aims of ecosystem service assessments were to quantify nature’s economic value and consequently showcase the importance of nature to human society [8]. Nevertheless, a straightforward relationship between ecosystem values and ecosystem conditions has yet to be found.

An alternative approach to assess regional ecosystem conditions, aided by GIS, was introduced in the 1980s and 1990s. Schaeffer et al. [9], Costanza [10], and Rapport et al. [11] proposed conceptual frameworks of ecosystem health (EH) and ecosystem vulnerability (degree of weak and low resilience to external stressors for ecosystem condition assessment). Many regional ecosystem condition assessments, supported by these frameworks, have been conducted for informed decision making (e.g., ecological management, planning/zoning, priority setting, and funding allocation), enhancing public awareness, participation, and consensus building, and monitoring policy responses [12]. However, there have been few reviews examining the progress of the assessment of ecosystem conditions at regional levels using this approach. Here, we evaluate the conceptual frameworks, methodology, indicator selection, weighting, index formulation, and prospects of this ecosystem study approach.

## 2. Frameworks for Assessing Regional Ecosystem Conditions

The myriad of attempts to evaluate ecosystem conditions indicate the challenges of completing a successful evaluation [12]. As opposed to assessing specific ecosystems, a variety of ecosystems are generally mosaicked within a region. Consequently, multiple indicators from a variety of perspectives are necessary for assessment because climatic, geological, hydrological, biological, and anthropogenic factors combine to determine regional ecological processes. As a result, regional ecosystem conditions have been examined from multiple comprehensive conceptual frameworks (e.g., EH, ecosystem vulnerability, and ecosystem security) in recent decades.

### 2.1. Ecosystem Health

EH is considered the basis of environmental management; a healthy ecosystem should provide sustainable services to society for current and future generations [10,11,13]. EH has been adopted as both an integrative concept institutionalized in national and international environmental management strategies, policy, and law and an approach for many projects and programs to evaluate, monitor, and restore ecological and human well-being [13,14]. The framework for ecosystem health assessment has evolved in recent decades. Originally, EH was proposed as a concept to evaluate three groups of primarily natural indicators: vigor (e.g., metabolism, biomass, and productivity), organization (e.g., biodiversity and interactions between system components), and resilience (the capacity to maintain structure and functions under stress) (e.g., [9,10]). More recently, the recognition of the importance of EH to human health and the connection between EH and ecosystem services make it necessary to incorporate ecosystem services in analyses [11,13]. It has been suggested to integrate socioeconomic and human health dimensions into the measurement of EH [11]. In this broad sense, EH resembles the concept of sustainability that includes environmental, social, and economic dimensions.

### 2.2. Ecological Vulnerability

As a concept originating from social science, ecological vulnerability is understood as the degree of weak resistance ecosystems and low resilience ecosystems to stressors [15,16]. Generally, ecological vulnerability can be evaluated from six general perspectives: exposure (to stressors), sensitivity (of the community to stressors), responses, resilience (the amount of change that a system can endure without changing its state), community structure and function, and adaptive capacity at different organizational levels (organism, population, community, ecosystem, and landscape) [15,17,18]. Similar to EH, ecological vulnerability analysis methodologies are more for specific ecosystems (e.g., shores and marine ecosystems) than for regions comprising a mosaic of ecosystems.

### 2.3. Ecological Security

The origin of ecological security resulted from expanding the definition of security: the impact of economic and social stability on environmental quality [19]. Ecosystem security refers to the security of natural and semi-natural ecosystems—a component of ecosystem health. Ecosystem (or environmental) security may be defined as the risks or vulnerabilities of losing ecosystem goods and services as well as the perception of those risks [20]. In addition, ecological security may resemble ecosystem health (i.e., characterize the health status of ecosystems, [21]). Although the meaning of ecological security can differ depending on the scale of assessment, it is the opposite of ecological vulnerability. As a result, there has been no widely recognized indicator system for the assessment; it may focus on any processes that mitigate regional ecosystem degradation and minimize adverse impacts on humans (e.g., [22]).

### 2.4. Environmental Sustainability

The aim of environmental management is to obtain sustainable ecosystem services for current and future generations. Consequently, environmental sustainability is the target of ecosystem condition monitoring and assessment and landscape management. In addition to these approaches for ecosystem condition assessment, sustainability itself has become an assessment tool to gauge the status of socio-economic development and ecosystem conditions. In general, sustainability has three dimensions: ecological/environmental, economic, and social. A hierarchically integrated index based on a variety of indicators is generally formulated to indicate degree of sustainability at global, national, and regional scales [23,24]. Some commonly used indices include the Human Development Index, the Happy Planet Index, the Wellbeing Index, the Sustainable Society Index, the Environmental Sustainability Index, and the Environmental Performance Index. Sustainability assessments at the national scale have been thoroughly examined in several reviews of the critical steps for formulating sustainability indices (e.g., [24,25,26]).

### 2.5. Identifying Environmentally/Ecologically Sensitive Areas

This conceptual framework pertains largely to environmental management, i.e., to evaluate the status of the ecosystem, very similar to the four previously described perspectives and is used to prioritize conservation/remediation areas for management purposes. Likewise, a series of indicators focused on biodiversity, vegetation condition, and landscape patterns are selected for this assessment [27].

## 3. Methodology

**Figure 1:**
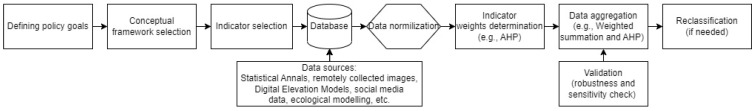
A general procedure of regional ecosystem condition assessment.

The general steps for conducting a regional ecosystem condition assessment are defining policy goals, selecting indicators based on a conceptual framework, selecting suitable weighting and aggregation techniques, and checking for robustness and sensitivity (Figure 1). The results may be reclassified to facilitate communications. The assessment is generally completed in a GIS setting. Indicating variables (or indicators) (e.g., land use and Normalized Difference Vegetation Index/NDVI) and MCAs are commonly adopted to reflect regional ecosystem conditions. Although they have been criticized for not including interactions among variables [1], the relative simplicity of the indicators is useful to communicate complex ideas for policy making or for public awareness [28,29]. 

### 3.1. Research Platforms and Data Sources

Regional ecosystem condition assessment is commonly conducted in a GIS setting. Currently, the most common GIS data format for ecosystem condition assessment is a raster format consisting of grid cells. One cell is the smallest unit of the layer, which corresponds to a prescribed area (e.g., 10 × 10 m^2^) of the Earth’s surface. Each cell has one value that indicates cell attributes (e.g., elevation, slope angle, land use type, and population density). It is relatively straightforward to aggregate indicators through weight summation.

Effective spatial data to determine the status of natural capital and anthropogenic impacts are required for the assessment of regional ecosystem conditions. By providing repeatable measurements from space, remotely sensed images have played significant roles in monitoring and assessing temporal and spatial ecosystem conditions, especially for providing indicators and inputs for ecological process modeling [30]. For example, species or dominant species mapping can be extracted from high spatial resolution optical images, images collected by active sensors, or combinations of multi-temporal images [4].

Many environmental/ecosystem indicators (e.g., land use and plant cover) can be extracted from remotely collected images at various resolutions for regional assessment (Table 1). Many indicators have been measured from ground-based sampling points, with models used to interpolate values for the unsampled landscape (e.g., atmospheric deposition of nitrogen and sulfur compounds). Other commonly used data include soil, forest, and species surveys/inventories. The availability of landscape metrics derived from remotely collected imagery and Digital Elevation Models (DEM) provides the opportunity to identify impacts of landscape patterns on ecological processes. Due to the lack of appropriate spatial data for many ecosystem functions and processes, modeled variables are frequently used as indicators for ecosystem condition assessments (e.g., [31,32]). It is practical to model biodiversity, the carbon cycle, primary production, soil erosion, and other indicators using DEM and remotely collected data [33,34,35].

The data for these assessments can be categorized into two groups: statistics collected within discrete administrative boundaries and spatially continuous data. Early regional assessments of ecosystem conditions were primarily based on administrative boundaries where many statistical data (e.g., gross domestic production (GDP), population density, and industrial wastewater treatment rates) within the boundary were collected (e.g., [39,44]). While the results from this type of assessment provide evidence for differences in general environmental/ecosystem conditions among administrative units (e.g., countries, provinces, and counties), spatial variation within the boundary may be lost or neglected. Only in recent years has there been more research using spatially continuous data (e.g., [38,45]).

### 3.2. Multi-Criteria Analysis (MCA)

The most common approach to assess regional ecosystem conditions is an MCA (also referred to as multi-criteria evaluation and multi-criterial decision analysis). Various elements (attributes) of ecosystems (e.g., slope, distance to a road, population density, and primary production) are used as the inputs for the assessment. Elements are sometimes standardized to a common value range to minimize the impacts of measurement metrics. Element weights are also commonly determined from literature values, experiments, or expert opinion. Finally, multiple criteria are combined to create a solution. Weight summation is the most popular approach for criteria combination in a GIS environment [46] and the AHP is a common method for weight determination and weight summation. 

#### 3.2.1. Indicator Categorization

The variety of ecosystem/environment components and intricate interactions among them make indicator selection challenging. A conceptual understanding of the regional ecosystem is critical to identify key elements and interactions among them that contribute to a sustainable ecosystem. Indicator categorization facilitates conceptual development, reductions in the degree of subjectivity in the indicator selection process, and results interpretation [24,47]. Vigor-Organization-Resilience (VOR) and PSR are two commonly adopted models for this purpose.

Originally, the VOR model was recommended for assessing specific ecosystems at different scales (Table 1). Spatially quantified indicators are collected to assess regional ecosystem conditions. The VOR framework has been criticized for underrepresenting driving forces (impacting factors) of ecosystem health and the mechanisms of ecosystem response [48]. Improving this model would include the ability to incorporate fundamental drivers of ecosystem transformation (e.g., demographic changes and income disparities) [49].

In contrast, the PSR framework integrates stressors (impacting factors), state of the environment or ecosystem measures (function, structure, or natural resources/services it provides), and societal responses (human response to changes to restore the environmental quality or prevent environmental degradation) [50]. PSR categories have been found to be more comprehensive in dealing with the limitation characteristics of the VOR model. As a result, some have integrated PSR and VOR frameworks to enhance ecosystem condition monitoring (e.g., [36,48]). Recently, advances have been made in evaluating ecosystem conditions with the PSR framework using remotely collected and other spatially continuous data (e.g., [38,45,51]).

Although the number of potential indicators that represent ecosystem condition and trends at the community and finer scales is normally large, the challenge is greater at the regional scale. Consequently, slightly different indicators are generally adopted at the regional scale. Commonly used vigor indicators include NDVI, NPP, and per capita GDP (Table 1). NDVI is the most common due to its connection to productivity and easy data access (e.g., [38,52]). Landscape metrics (e.g., landscape diversity index and average patch size) and the percentage of tertiary industry in GDP are common organizational (structural) indicators. An ecological elasticity indicator, determined using the landscape diversity index and resilience of different landscape types [53], is widely used to represent resilience generally. Common pressure indicators are population density, per capita arable land area, distance to roads, distance to villages, slope angles, and pollutant concentrations in water, air, or soil. Note that one indicator may be applied to different categories. For example, NPP, a commonly used vigor indicator, may indicate the ecological recovery capacity (resilience) (e.g., [54]). Trade-offs are common because of the challenges in collecting indicators at the regional scale, leading to situations such as biodiversity being represented by vegetation community diversity information extracted from Landsat imagery (e.g., [55]).

#### 3.2.2. Indicator Selection

Regional variation, system complexity and uncertainty, and data availability make it challenging to use a uniform set of indicators across regions. Generally, one index combining indicators of the ecosystem condition (structure, function, and processes), economy, ecosystem services, landscape pattern, and land use is formulated to represent the ecosystem condition. This holistic index approach, an operable tool for understanding regional variation and environmental management, is currently the norm for regional ecosystem assessment [36]. Indicators available from Statistics Annals (or Statistical Yearbook) (e.g., population density), and others that can be measured using remote sensing tools, dominate. There had been a slow transition from political boundaries in the early years to spatially explicit assessments.

In general, indicators selected for assessment should be efficient in measuring ecosystem degradation from multiple perspectives. Many selection criteria have been proposed (e.g., [1]), including ease of measurement, cost effectiveness, sensitivity to ecological stress, reliability and availability of historical data, scale appropriateness, and process-orientation, as well as including the possibility of deriving political parameters [1,56]. Furthermore, it is advisable to use widely recognized indicators with clearly articulated justification [24]. Finally, the connection of these indicators to the assessment framework should be clarified and defensible. Consulting experts in the field may be helpful during indicator selection and modeling [55,57]. However, trade-offs between desirable characteristics, costs, and quality of information are inevitable when choosing indicators for assessment.

Indicators should be selected to maximize unique and relevant information while minimizing redundant information [47]. One of the challenges of using multiple indicators is identifying and managing correlations among them. Correlations among indicators may be examined to avoid multidimensionality problems. Principal Component Analysis (PCA) may be applied to aggregate data dimensions (e.g., [22]); nevertheless, care should be taken when including more indicators. There have been cases where two strongly correlated indicators have been applied to weight a single category (e.g., [58]). Rationale should be given regarding the use of highly correlated indicators in analyses and the impacts on assessment results. Spatial autocorrelation in the dataset should be determined and correlation impacts on results should be mitigated and reported [55,57]. Geostatistical tools, specifically semivariance, can be used to identify the range of spatial dependence. Moran’s I is a useful tool to quantify the magnitude of autocorrelation.

Data normalization may help to avoid potential problems in aggregating indicators at different measurement scales and with different units. In many cases, collected data for multiple indicators may need to be normalized/standardized to the same data range (e.g., 0–1 or 0–100) to accommodate for respective reference values (the benchmark) [11,59,60]. Similarly, data should be transformed to facilitate appropriate comparisons based on percentage differences. Commonly used methods to determine threshold/benchmarks include local investigations, historical references, comparisons, national standards, relevant research results, public participation, and expert judgments [22,51]. Alternatively, indicators can be standardized by the distance from the mean, the standard deviation from the mean, and the distance from the best and worst performers [56]. Linear, square root, logit, or logarithmic transformations can be used in data normalization, with linear transformations being the most frequently used method [61]. Finally, fuzzy measures have been suggested as the preferred choice for indicator standardization [25]. However, this is yet to be adequately tested.

#### 3.2.3. Combining Indicators

The fact that multiple indicators are needed to identify ecosystem conditions from multiple perspectives suggests that determining how weighting contributes to combining indicators to form a holistic index remains a challenge; the usefulness of the index is defined by the weighting and combination method used [62]. Constructing a combined indicator is based on the decision rules upon the request of decision makers and the public; one value or a limited number of values which include multidimensional phenomena is easier to understand than an array of data [26]. Critical issues to be considered for indicator combination are indicator weights and combination methods.

Determining variable weighting is generally critical to calculate a holistic index; assessment results are sensitive to the weights applied. The most common determination method for MCA is the pairwise comparison weighting procedure [61], in which expert opinions are generally incorporated into the process. However, it is not unusual to see equal weights (or an average of all indicators) used in sustainability assessments; about half of assessments used equal weighting methods [62]. Equal weights were justified by the lack of evidence for the contrary.

Although weighted summation and the analytical hierarchy process (AHP) are the mostly commonly applied methods for GIS-based MCA, artificial neural networks (ANN), genetic algorithms (GA), and other models (e.g., the fuzzy synthetic assessment model, the attribute theory model, and set pair analysis) may also be applied to integrate indicators [63]. Costanza [10] proposed an operational definition of an ecosystem health index (HI): HI = Vigor × Organization × Resilience. There have been cases where the cubic root of the production was used to calculate the index (e.g., [52]), but indicator combinations may also be determined through spatial principal component analysis (e.g., [64,65,66]). Nevertheless, care must be taken when integrating values of ratio and interval scales; it may be impossible to aggregate them in a meaningful way [56].

The scale at which data were collected may impact assessment results. Ideally, multiple indicators should be collected simultaneously. However, due to the lack of regional data, data at national (e.g., [38]) or global scales (e.g., [67]) may be adapted for regional analyses. Care must be taken regarding scale, however, with specific attention paid to how it may impact data (quantitative and positional) accuracy and the assessment results. It is expected that this data restriction issue will be eventually resolved as more spatial data becomes freely available when more government and non-governmental agencies open online sharing at no cost. This should trigger enhanced research in evaluating the sensitivity of indicator systems to scale selection, developing and refining methods for combing multiple indicators to a common scale, and identifying critical limits and thresholds [12]. Spatial data should additionally be verified for their quality to conduct the assessment. Population density and crop production are important pressure (or vigor) indicators for ecosystem condition assessment. To create spatially continuous data (e.g., grain production and population density) based on statistical and remotely collected data, coefficients are normally assigned to complete the calculation (e.g., [38,67]). However, validation has not generally been conducted (or reported) for data quality assurance.

## 4. Discussion and Prospects

A primary challenge for regional environmental assessments lies in the integration of information from select but diverse indicators into an overall ranking of environmental condition [58]. This results in identifiable limitations to conducting regional ecosystem condition assessments, e.g., lack of essential synoptic data for a region, failure to integrate across natural and human dimensions, and the lack of appropriate analytical methods capable of capturing the high degree of complexity inherent in these regional systems [13,33]. The potential research areas for ecosystem condition assessment suggested by Costanza [13] remain the same: integration of socio-cultural variables in assessments and more operative indicators primarily based on remote sensing technology. Furthermore, it is expected that big data will enrich information sources for assessments. In addition, validation of both spatial data and assessment results should be a necessary component considering the challenges and uncertainties involved in data collection and analyses.

### 4.1. Incorporating More Socio-Economic Indicators Reflecting Human Needs and the Interactions between Humans and the Environment

To date, no assessment has effectively integrated ecological and socio-economic components and their interactions at a regional scale. It has proven challenging to collect socio-economic indicators (e.g., income disparity and consumerism) at a regional scale, which has largely prevented their incorporation to date [49]. Natural or anthropogenic responses are often difficult to quantify because of the complexity of the human–environment system and decisions regarding responses. It is, however, necessary to explore common indicators to reflect human needs and the interactions between humans and the environment [49]. The links between socioeconomic drivers, urbanization patterns, and their influences on ecosystem conditions need to be investigated during the assessment process. Indeed, the proposed content of EH assessment proposed by Rapport et al. [11] is similar to sustainability assessments where natural, economic, and social dimensions are involved.

To tackle the challenges of combining indicators, new indices such as biocultural diversity, human development, and quality of life should be developed [49]. Social big data (crowdsourced), in combination with machine learning, may well be critical in understanding human–environment interactions [68]. For example, emotional expressions regarding the environment may be extracted with the aid of machine learning technologies [69]. Based on survey data and satellite imagery, a machine learning model with a convolutional neural network was applied to estimate consumption expenditure and asset wealth [70]. This approach may be promising to scale up point survey data to become spatially continuous. More details about socio-economic indicators (e.g., urbanization, GDP, population density, and poverty) are presented in the following section.

### 4.2. Incorporating Spatial Big Data and Machine Learning into the Assessment

Although data availability strongly impacts the selection of indicators for assessments, big data and machine learning technology can potentially change the status quo. Advances in data generalization in high resolution remote sensing are moving at unprecedented speeds, as are smart information and communication technology enabled by the Internet of Things (IoT), cloud computing, and machine to machine infrastructure [71]. The characteristics of big data—high volume, variety, velocity, and variability—may facilitate the investigation of regional ecosystem dynamics. Currently, environmental big data may come from any or all of Earth Observation Systems, ground-based monitoring networks and the IoT, simulated Earth system data and crowdsourced data from social media, and citizen science [71].

Further advances in remote sensing technology make it possible to monitor ground conditions for large areas. The availability of satellite images from a variety of sensors and new techniques for data processing (e.g., data fusion and data assimilation) may present a new horizon for ecosystem condition assessments. For example, the Advanced Topographic Laser Altimeter System (ATLAS) onboard the ICESAT-2 platform provides continuous height measurements for ecosystem monitoring. LANDSAT, MODIS, and SPOT-VEGETATOIN NDVI have been widely adopted to represent resilience and vigor. Soil Moisture Active Passive (SMAP) satellite missions resulted in surface and root zone soil moisture estimates, along with other geophysical estimates, with a temporal resolution of 3 h and spatial resolution of 9 km [72], which may be applied in regional scale assessments.

Remotely sensed nighttime light (NTL) satellite imagery (e.g., Defense Meteorological Satellite Program—Operational Linescan System (DMSP-OLS) and the Visible Infrared Imaging Radiometer Suite (VIIRS) of Suomi National Polar-orbiting Partnership) is able to efficiently extract information regarding light pollution, urbanization, economic activities (e.g., GDP), population density, and poverty [73]. The positive correlation between population, economy, and NTL makes it possible to extract data of regional inequality, electricity consumption and access patterns, household level income, variation in freight traffic, human impacts on the environment, and conservation [73]. The combination of NTL and optical imagery (e.g., MODIS and LANDSAT) may also facilitate enhanced extraction of socio-economic indicators for ecosystem condition assessments. Furthermore, a Developing Relative Spatial Poverty Index has been proposed using derivatives from satellite imagery and GIS [74].

Recent progress in hyperspectral remote sensing (collecting images with a multitude of narrow spectral bands) has made it practical to map plant traits (e.g., chlorophyll, nitrogen, and phosphorous) from remotely collected images [75] for regional ecosystem condition assessments. It is currently possible to extract crop carbon content and biomass by combining hyperspectral data, radiative transfer modeling, and machine learning algorithms [76]. Furthermore, the spatial resolution of remotely collected images may influence assessment uncertainty. For study areas that require both relatively high temporal and spatial resolution, image fusion (i.e., the fusion of high spatial/low temporal resolution and low spatial/high spatial resolution images) may be a practical solution.

Another potentially usable data source for regional assessments is crowdsourcing (big data). Where there are no authoritative spatial data available, crowdsourced geographic data may be able to fill the gap [77]. Volunteered geographical information (VGI) and social media geographic information (SMGI) may be incorporated into the assessment if the minimum quality thresholds are met [78]. Spatial data extracted from location-based social media data have been shown to be comparable or even better than data derived from NTL imagery in identifying socioeconomic activities [79]. VGI may be used to update/quality check authoritative spatial data. For example, OpenStreetMap, the world’s largest crowdsourcing platform, provides data on habitats, biodiversity, and networks that may be available at urban scales [78]. Point patterns of frequently photographed places by tourists can be used to indicate landscape attractiveness and preference.

Recent developments in the incorporation of cloud-based geospatial platforms (e.g., Google Earth Engine) and machine learning techniques also facilitate data collection, processing, and regional assessments. Machine learning technology (e.g., artificial neural networks, random forests, and support vector machines) and regression or classification (supervised or unsupervised) of ecosystems have been applied to extract indicators for environmental assessments for their capability to address the challenges of data gaps and uncertainty and to connect social and ecological factors [80,81,82]. For example, airborne particulates were successfully mapped by integrating data from ground measurement stations and machine learning techniques [80]. A machine-learning-enabled land use model was better than a land use regression model to map particulates at a regional scale [83].

### 4.3. Examining Uncertainty in Spatial Data and the Robustness of the Assessment

It is unlikely that a single assessment can fully identify ecosystem (or human–environment interactions) dynamics with complete certainty [49]. There are cases where ecosystem conditions were nevertheless degraded after stressors were removed (e.g., desertification of the arid grasslands in Southwestern USA, [49]). Consequently, uncertainties should be part of any assessment. Studies should identify and report the challenges faced and potential future directions.

Uncertainty in assessment results originates primarily from data and combination rules [77]. Field collected data (through interpolation), statistics data, data extracted from remotely collected images, and modeled data have been commonly applied in ecosystem condition assessments. However, data quality for a region should be identified and reported. In many cases, there are multiple data sources for regional ecosystem assessments and quality control for crowdsourced or VGI data remains a challenge. It is preferable that a crowdsourcing system include measures and data specifications to assure user input accuracy and that tools implemented allow volunteers to monitor data content [84]. Care should be taken when combining these data for further analysis, for example, it should be ensured that the data match well positionally. Matching remotely collected data with administrative statistics may be challenging; aggregation of remotely collected data may reduce data precision [85].

To examine the uncertainty of regional assessments, it is preferable that both data uncertainty and sensitivity analyses are conducted [77]. Uncertainty analyses quantify the variability of the results caused by variation among indicators. Sensitivity analyses quantify how assessment variability can be allocated to parameter uncertainty. For regional ecosystem condition assessments, sensitivity analyses are often conducted to examine indicator weights impacts. While the weight assigned to a variable can be interpreted as the importance of the indicator to the holistic index, variances and correlations among variables may prevent the weights from corresponding to the indicator’s importance [86]. Exploratory tools have been developed (e.g., [86]) to identify the impacts of weights on the index and to refine the indicator. However, few peer-reviewed articles have reported some form of sensitivity analysis. Consequently, we suggest that efforts be made to better identify the effects of weight on index formation in the study of ecosystem condition assessments. There are several methods (e.g., fuzzy AHP, the membership function, and Monte Carlo simulation) to address uncertainty issues [67].

### 4.4. Exploring Capability of MCA Methods for Regional Ecosystem Assessments

Applying different MCA methods for one assessment task can result in variable outcomes (e.g., [87]). Consequently, a comparative analysis of the impacts of adopting different methods may give insights into sensitivity, methods, and parameters of the results and to determine whether methods are compatible or complementary [77]. Spatial weights, the critical parameters for MCA with geographically varying outcomes, may be an effective way to explore the applicability of regional ecosystem condition assessments.

We have identified three new directions for future methodologies, mirroring those of Malczewski and Jankowski [77]: comparison (of different methods), augmentation (improving a method by incorporating elements of other methods), and integration (new method development through integrating the existing methods). We suggest that it is important that the most recent advances in quantitative techniques and tools be incorporated in ecosystem assessments. The nature of the assessment dictates that it is a multidisciplinary effort; collaboration between ecologists, geospatial analysts, statisticians, and researchers in any relevant disciplines is critical in advancing regional ecosystem condition assessments.

One trend in regional ecosystem condition assessments is the hybridization of MCA techniques, fuzzy methods, and other emerging technologies [25,77]. Although AHP has been widely used for pairwise comparison to establish a matrix of relationships among indicators, it has been criticized for its inability to deal with the inherent uncertainty relevant to quantifying decision makers’ perceptions [22,41]. Consequently, fuzzy AHP has been proposed to capture the subjectivity of a human’s appraisal in a complex, multi-criteria justification process. Fuzzy (e.g., [22,88]) and gray (e.g., [89,90]) AHPs have been applied to examine ecosystem health, ecosystem security, and ecosystem vulnerability. Similarly, the combination of PCA and the Analytic Network Process (ANP) to integrate environmental indicators has been shown to be superior to other methods (e.g., [91]). Similar to the AHP and its variants, there are several other approaches that can integrate the process of weighting and combining (e.g., attribute theory model, catastrophe progression method, unobserved component models, and budget allocation) [22,62]. Further research is needed to examine the efficiency of these methods for regional assessments.

### 4.5. Identifying Regional Ecosystem Directions of Change

Ecosystems continually evolve. Trends in ecosystem changes should be one consideration of environmental management and decision making. Future direction/trends may be uncovered by identifying ecosystem conditions in the long term (5 years or longer). This helps remove the impacts of natural ecosystem dynamics (e.g., phenology) on assessment results and may help to delineate and understand regional abnormality and sudden and non-linear changes [92]. As a result, regular assessment of regional ecosystem conditions should be the norm, not the exception. Surprisingly, few studies have incorporated a time stamp, that we suspect may be due to the high maintenance cost of long-term studies [92]. The ever-increasing magnitude of open source and higher resolution spatial data and geoprocessing platforms may help alleviate this. As we have identified, research can be facilitated by collaborations among ecologists, remote sensing scientists, data analysts, and other relevant disciplines.

## 5. Conclusions

This review examined the involvement of MCA in comprehensively assessing regional ecosystem conditions from concepts to applications in the last four decades. Generally, multiple factors are needed to identify ecosystem conditions. Steps to be followed to complete an assessment are indicator categorization, indicator selection, and indicator combination. The most common methods to combine indicators are weighted summation and the AHP. In the future, it is very likely that more socio-economic indicators (regional inequality, electricity consumption and access patterns, household level income, and human impacts on the environment) will be included in assessments thanks to advances in spatial big data and machine learning algorithms. Likewise, uncertainty and sensitiveness analyses should be included in future research. It is likely that MCA methods may be compared, augmented, and integrated to advance regional ecosystem assessments. It is also critical that regular regional ecosystem condition assessments are conducted to identify general change trends.

## Figures and Tables

**Table 1:** Selected spatially continuous indicator candidates for ecosystem condition assessments.

Perspective of the Regional Ecosystem	Indicator	Relevance and Data Sources	Example References
Vigor	Carbon density	Modeled (empirical or process-based) primary production	[36]
	NDVI	“Greenness” of the land surface from remotely collected data	[37]
	Ecological service	Products/services provided by ecosystem, collected by data from a myriad of sources	[38]
Structure	Landscape diversity index	A surrogate indicator for species diversity and landscape resilience, may be extracted from images or DEM	[39]
	DEM	Derivation of slope angle, soil moisture, and soil erosion risk	[40]
Pressure	Road network	Accessibility and proximity to pollutants/disturbance relevant to transportation. Vector data, generally available from governmental agencies	[41]
	Surface geology	Geological conditions (e.g., parent materials) may indicate soil and vegetation conditions. Vector data, generally available from governmental and NGO agencies	[42]
	Population density	Anthropogenic pressure on ecosystems, data generally available from governmental agencies	[38]
	Air/water/land quality indicators	Stress on the ecosystem, generally available from governmental databases, may be modeled to provide regional conditions	[43]

## Data Availability

No new data were created or analyzed in this study. Data sharing is not applicable to this article.
